# Validation of the sibling acceptance questionnaire among typically—developing emerging adult siblings of individuals with disabilities

**DOI:** 10.3389/fpsyg.2026.1764034

**Published:** 2026-02-20

**Authors:** Raaya Alon, Or Catz

**Affiliations:** 1 Department of Special Education, Michlalah Jerusalem College, Jerusalem, Israel; 2 Department of Psychology, Ashkelon Academic College, Ashkelon, Israel

**Keywords:** developmental disabilities, factor analysis, psychometric validation, sibling acceptance, typically-developing siblings

## Abstract

Sibling acceptance is a central component of sibling relationships, including when one sibling has a developmental disability, shaping the quality, stability, and long-term functioning of the family. Despite its importance, sibling acceptance has received little systematic quantitative attention, and validated measures remain scarce. This study validated the Sibling Acceptance Questionnaire among typically-developing emerging adult siblings of individuals with developmental disabilities. The original instrument, developed by Brenner to assess parental acceptance, was adapted for siblings but had not undergone rigorous psychometric evaluation. To do so, data were collected from 854 siblings of individuals with autism spectrum disorder (ASD) or Down syndrome. Exploratory and confirmatory factor analyses supported a three-factor structure: perceived burden and limitation, openness to sharing, and perceptions of parental attention. Internal consistency across factors indicated reliable measurement. Content validity was further supported by significant associations between functional independence and both burden/limitation and openness to sharing, but not parental attention. By establishing the reliability and validity of this instrument, the study addresses a methodological gap and provides researchers and practitioners with a valuable tool for advancing research and interventions that promote sibling relationships, family functioning, and the well being of siblings with and without disabilities.

## 1 Introduction

This study examines the validity of the Sibling Acceptance Questionnaire among typically- developing siblings of individuals with disabilities. The original instrument, developed by Brenner (1979), was designed to assess parental acceptance of a child with a disability, but the concept of sibling acceptance has received little systematic and quantitative attention, despite its centrality to sibling relationships. Only limited work has been carried out to establish reliable quantitative tools for assessing sibling acceptance. This gap is notable, as sibling acceptance represents a key construct in sibling dynamics, particularly when one sibling has a developmental disability such as autism spectrum disorder (ASD) or Down Syndrome. Acceptance influences both the quality and stability of sibling ties and may carry long-term implications for family functioning and caregiving roles (Correia and Seabra-Santos, 2021; Trew, 2025). The absence of validated instruments in this domain restricts both research and the development of targeted interventions. To address this gap, the present study examined the factorial validity of the Sibling Acceptance Questionnaire using exploratory and confirmatory factor analyses and assessed its content validity in a large sample that provides a solid basis for psychometric validation.

### 1.1 Sibling acceptance

Acceptance is conceptualized as a multidimensional construct that reflects the recognition that characteristics associated with disability form an integral part of a person's identity, that is, they accept the individual “as they are” (Alon, 2025a; Johnson et al., 2020). Acceptance develops dynamically as family members deepen their understanding of the diagnosis and the needs of the individual with the disability (Corsano et al., 2017; Mokoena and Kern, 2022). Emotional, cognitive, and social adjustments are necessary to reach acceptance, reflecting a complex psychological process (Johnson et al., 2020; Petalas et al., 2012). Acceptance is often considered alongside complementary constructs used in the sibling literature, such as emotional closeness/warmth, ambivalence, and caregiving-related orientations (Leane, 2020; Calio and Higgins-D'Alessandro, 2021; Travers and Carter, 2020). Nonetheless, the complexity of the process and multidimensional nature of the construct emphasize the need to investigate acceptance as a potentially independent dimension, rather than as a derivative of other relational features.

Acceptance constitutes a central component of sibling relationships when one sibling has a disability. Importantly, acceptance does not simply represent the absence of conflict but is distinctly related to expressions of warmth and closeness (Tomeny et al., 2017). Research shows that emerging adults who report higher levels of acceptance toward their sibling with a disability also describe closer ties and more varied shared activities, reflecting warmth, and voluntary engagement (Travers and Carter, 2020). In addition, acceptance has been found to underlie the desire of typically-developing siblings to maintain good relationships with their siblings with disabilities, despite communication barriers and daily challenges with their siblings with disabilities (Wright et al., 2024).

Acceptance can foster voluntary closeness and contribute to the long-term sustainability of family roles (Noonan and Wilson, 2018; Tozer and Atkin, 2015; Tozer et al., 2013). Acceptance also has important implications for the psychological well being of typically-developing siblings. Higher acceptance has been linked to better adjustment, greater optimism, and a stronger sense of coherence (Alon, 2025a; Múries-Cantán et al., 2023). Conversely, lower levels of acceptance, particularly in families of individuals with significant functional impairments, have been associated with increased risk for mental and physical health difficulties among typically-developing siblings (Brennan et al., 2023).

### 1.2 Emerging adulthood

Emerging adulthood is conceptualized as a distinct developmental period, typically spanning the late teens through the 20s, during which individuals transition gradually from adolescence to full adult roles (Arnett, 2015). This stage is characterized by a sense of being “in-between”: young people are no longer adolescents, yet they have not fully attained stable markers of adulthood such as long-term partnership, stable employment, or parenthood (Arnett, 2000). Emerging adulthood is often marked by intensive exploration of identity in the domains of work, relationships and worldview, frequent changes in education, work and residence, and a heightened emphasis on autonomy, self-focus, and personal choice (Nelson and McNamara Barry, 2005). Alongside the opportunities for growth and self-discovery, emerging adulthood can also involve considerable uncertainty, stress related to long-term decision making, and a lack of stability and security in daily life (Gungordu et al., 2022).

For siblings of individuals with disabilities, the challenges and tasks of emerging adulthood may become particularly salient and complex (Murrin et al., 2021). These siblings must negotiate the developmental tasks of separation–individuation and identity formation while simultaneously managing ongoing emotional bonds, responsibilities, and concerns related to their brother or sister with a disability (Laghi et al., 2018). They may experience heightened worry about the future care of their sibling, the aging of their parents, and their own anticipated role as potential primary caregivers, all of which can shape decisions regarding education, employment, place of residence, and intimate relationships (Iannuzzi et al., 2022). As a result, emerging adulthood for these siblings may be characterized by ambivalence—combining feelings of love, commitment, and pride with experiences of burden, guilt, and emotional strain—as they attempt to integrate family obligations with their own life paths (Calio and Higgins-D'Alessandro, 2021). During emerging adulthood, acceptance becomes particularly critical, as siblings increasingly assume caregiving responsibilities as parents age (Sharabi and Siman Tov, 2022). Positive attitudes grounded in acceptance predict greater willingness to provide support, while higher levels of acceptance are associated with sustained and active involvement of the typically-developing sibling in the life of the sibling with a disability (Leane, 2020; Tomeny et al., 2017).

### 1.3 Sibling functioning and sibling acceptance

Research on siblings of individuals with disabilities indicates that the functional and behavioral characteristics of the brother or sister with a disability are closely tied to siblings' emotional burden and the quality of their relationships. Lee et al. (2019) found that maladaptive behaviors of the individuals with a disability, particularly asocial behaviors such as uncooperativeness or socially inappropriate behavior, were positively associated with typically-developing siblings' caregiving demands, including feelings of stress, emotional exhaustion, and physical fatigue. These associations were especially pronounced at the most severe levels of maladaptive behavior and are consistent with previous findings linking higher levels of such behaviors to more negative sibling relationships (e.g., Orsmond et al., 2009). In contrast, functional ability in day-to-day activities was not significantly associated with caregiving demands, suggesting that behavioral functioning may have a greater impact than basic daily living skills on siblings' subjective burden.

Studies by Alon (2022, 2025a),b examined the perceived independent functioning of individuals with Down syndrome (DS) or autism spectrum disorder (ASD) as a key factor in sibling acceptance. Perceived independent functioning refers to the extent to which the sibling with a disability is seen as able to manage daily and community activities with limited need for assistance; higher perceived independence indicates reduced reliance on family members for everyday support, whereas lower perceived independence indicates greater dependence and a heavier ongoing and future caregiving burden for the typically-developing sibling (Alon, 2022; Heller and Kramer, 2009). Alon (2022, 2025a),b showed that perceived independent functioning differentiated levels of acceptance, such that typically-developing siblings reported higher acceptance when the sibling was perceived as more independent, and perceived independent functioning emerged as a significant predictor in regression models of acceptance. Siblings of individuals with ASD who were perceived as having lower independent functioning reported lower acceptance (Alon, 2022, 2025a,b). These findings indicate that siblings' perceptions of the functioning and independent capabilities of their brother or sister with a disability play a central role in determining siblings' levels of acceptance and attitudes.

### 1.4 The current study

Overall, the above-reviewed literature identifies sibling acceptance as a complex, multidimensional construct with significant implications for relationship quality, psychological well being, and long-term family roles (e.g., Múries-Cantán et al., 2023). Despite growing scholarly attention, systematic quantitative assessment of sibling acceptance remains limited. Much of the existing research has relied on small samples (e.g., Moyson and Roeyers, 2012; Yoo and Lee, 2023), or has focused primarily on childhood (e.g., Aksoy and Yildirim, 2008), leaving emerging adulthood insufficiently studied. This gap emphasizes the need for a validated tool capable of reliably assessing sibling acceptance across diverse populations and levels of functional ability. As such, the current study sought to validate the Hebrew adaptation of the Sibling Acceptance Questionnaire among typically-developing siblings of individuals with developmental disabilities. Building on Brenner's original parental acceptance measure (Brenner, 1979), and its later adaptation for siblings (Koenig-Gorfinkel, 2004), this study aimed to examine whether the instrument demonstrates satisfactory psychometric properties when administered to a large and diverse sibling population that included siblings of individuals with ASD and siblings of individuals with DS.

The study objectives were to evaluate the factorial structure of the questionnaire using exploratory and confirmatory factor analyses, to assess content validity by examining the relationship between the functional independence of the sibling with the disability and the acceptance scores reported by the participating siblings, and to establish the internal consistency reliability of the adapted instrument. In line with these aims, the central research question was whether the validation process would demonstrate that the questionnaire is both reliable and valid, and whether the items retained after analysis would form coherent and meaningful factors that adequately represent the construct of sibling acceptance.

## 2 Methods

### 2.1 Participants

The study included 854 participants, with similar representation between siblings of individuals with ASD (56.1%) and siblings of individuals with Down Syndrome (43.9%). The sample was predominantly female (68%), while their siblings with disabilities were predominantly male (63.5%). The vast majority of the participants were single (64.8%), and about a third were married (32.1%). Ninety percent of participants reported being from a medium (54.1%) or high (36.4%) socioeconomic status. Participants ranged in age from 18 to 28 years (*M* = 21.93, SD = 2.95), while their siblings with disabilities ranged from 1 to 43 years (*M* = 16.35, SD = 6.84). Of the siblings with the disabilities, about half had a moderate level of functionality (54.1%), and a third had a high functionality level (36.4%). Table 1 presents the demographic characteristics of participants and their siblings with disabilities.

**Table 1:** Description of participants and their siblings with disabilities.

**Variables**	**Categories**	***n* (%)**
**Participating siblings**		
Gender	Male	271 (31.8%)
	Female	581 (68.0%)
	Missing	2 (0.3%)
Family status	Single	553 (64.8%)
	Married	274 (32.1%)
	Separated	20 (2.3%)
	Other	3 (0.4%)
	Missing	4 (0.4%)
Religiosity	Secular and traditional	191 (22.4%)
	Orthodox	407 (47.6%)
	Ultra—orthodox	218 (25.5%)
	Other	29 (3.4%)
	Missing	9 (1.1%)
SES	High	311 (36.4%)
	Moderate	462 (54.1%)
	Low	80 (9.4%)
	Missing	1 (0.1%)
**Siblings with the disability**		
Gender	Male	542 (63.5%)
	Female	311 (36.4%)
	Missing	1 (0.1%)
Disability	Autism	479 (56.1%)
	DS	375 (43.9%)
Functionality level	High	311 (36.4%)
	Moderate	462 (54.1%)
	Low	80 (9.4%)
	Missing	1 (0.1%)

### 2.2 Measures

#### 2.2.1 Sibling acceptance questionnaire

The present study utilized the Sibling Acceptance Questionnaire (Koenig-Gorfinkel, 2004), which took the original Hebrew scale (Brenner, 1979) for parents and adapted it to examine sibling acceptance of a child with a disability. The questionnaire consists of 25 items assessing the typically-developing sibling's attitudes toward their brother or sister with a disability. Items addressed domains such as openness about the disability, perceived family burden, and willingness to engage socially with the sibling (e.g., “The sibling with the disability limits the social life of all family members”; “In my opinion, it is better not to take a sibling with a disability to visit relatives, friends, or neighbors because they are burdensome”). Responses were given on a 5-point Likert scale ranging from 1 = strongly disagree to 5 = strongly agree, with higher scores indicating greater acceptance (see Appendix A for all items).

#### 2.2.2 Demographic questionnaire

A demographic questionnaire, developed by the principal investigator specifically for the purposes of this study, comprised 16 items and was divided into two sections. The first section collected information about the participating sibling, including age, sex, education, and related characteristics. The second section addressed the sibling with a disability, covering age, sex, level of independent functioning, and residential status (living at home or elsewhere). Independent functioning was assessed through participants' subjective evaluations of their sibling's abilities in everyday activities (dressing, eating, bathing, and toileting) as well as in community activities (using public transportation, grocery shopping, seeking help when needed, and recognizing potential dangers).

### 2.3 Procedure

The study received approval from the “Michlalah Jerusalem College Academic Institutional Ethics Committee”. Invitations describing the aims and procedures of the study were distributed through targeted WhatsApp and Facebook groups dedicated to siblings of individuals with ASD or Down Syndrome. Participants were also asked to share the questionnaire with other eligible siblings in a snowball fashion. Respondents who expressed interest received a digital version of the questionnaire and provided informed consent in accordance with ethical standards, with full assurance of anonymity. In total, 854 siblings completed the questionnaire and met the study's inclusion criteria. No compensation was offered.

### 2.4 Data analysis

Statistical analyses were conducted utilizing SPSS version 28 and R statistical software (version 4.3.3) with the “Lavaan” package. Demographic variables, individual scale items, and factor-level descriptive statistics were computed. Internal consistency reliability was examined using Cronbach's alpha, with values exceeding 0.70 indicating satisfactory reliability. To examine construct validity, confirmatory factor analysis (CFA) was employed to assess the adequacy of model fit. Model fit evaluation incorporated multiple indices: chi-square test statistics, root mean square error of approximation (RMSEA; acceptable fit < 0.08, excellent fit < 0.05), standardized root mean residual (SRMR; acceptable fit < 0.08, excellent fit < 0.05), comparative fit index (CFI; acceptable fit > 0.90, excellent fit > 0.95), and Tucker-Lewis index (TLI; acceptable fit > 0.90, excellent fit > 0.95). Although values above 0.70 are typically considered satisfactory, slightly lower values may be expected for brief subscales with few items; therefore, alpha values in the mid- 0.60s were interpreted cautiously as acceptable for early-stage validation and were discussed as an area for refinement.

## 3 Results

### 3.1 Exploratory factor analysis

We conducted an initial content screening and marked statements that were formulated as theoretical statements or declarations and less related to the sibling's feelings or perceptions, and which were theoretically less directly related to acceptance. Then we conducted an EFA on all items of the questionnaire. Based on the factor analysis, statements that had high loadings on several factors, as well as statements that had low loadings on all factors, were removed from the questionnaire, after their content was examined. For example, item 18, on planning household activities around the interests of the child with the disability, did not distinguish was loaded on several factors, without distinguishing between them. Items 15 (parents should not discriminate toward or against the child with the disability) and items 24 (feeling alienation toward the brother/sister with the disability) were in opposite directions on one factor, while items 5 (some argue that an individual with a disability will always have the disability and need to be accepted as such) and 6 (some people think that it's natural that a child with a disability cause parents to have less patience with them) were separate factors. The EFA reinforced our initial analysis that certain items needed to be removed either for content or the statistical strength of the factor loadings. As such, the analysis of the questionnaire took place on 14 items: 1, 2, 3, 4, 7, 9, 10, 11, 12, 13, 14, 19, 21, and 22, representing the most reliable indicators of sibling acceptance. Internal consistency for this refined version was satisfactory (Cronbach's α = 0.87). Following this, new sequential numbers were assigned to the items, from 1 to 14.

Given that all data were obtained via self-report measures at one time point, statistical procedures were implemented to verify that the dataset was not characterized by a single general factor. To evaluate potential common method bias, Harman's single-factor test was employed (Podsakoff et al., 2003). An unrotated principal components analysis was performed, incorporating all 14 items from the study measures. Findings revealed that the initial factor explained 36.97% of the total variance, falling below the 50% criterion that would suggest significant common method bias.

To examine the underlying factor structure characterizing sibling acceptance of siblings with disabilities, an EFA was conducted without constraining the number of factors, employing Varimax rotation on the sibling acceptance questionnaire. The analysis results, presented in Table 2, revealed a three-factor solution accounting for 56.83% of the total variance.

**Table 2:** Factor analysis and factor loadings of the sibling acceptance questionnaire.

**Items**	**Factor 1—burden and** **limitation**	**Factor 2—openness to** **sharing**	**Factor 3—parental** **attention**
**Item 3** [3]—Disability disrupts siblings' lives	**0.803**	0.005	0.132
**Item 9** [6]—Limits family social life	**0.769**	0.078	−0.019
**Item 2** [2]—Avoiding outings with sibling	**0.738**	0.122	0.231
**Item 7** [5]—Disability worsens family life	**0.701**	0.208	0.170
**Item 19** [12]—Avoiding strangers with sibling	**0.663**	0.167	0.225
**Item 10** [7]—Not taking sibling to visits	**0.592**	0.215	**0.372**
**Item 1** [1]—Hiding disability from relatives	**0.522**	0.293	0.249
**Item 11** [8]—Openly sharing sibling's difficulties	0.224	**0.776**	0.115
**Item 4** [4]—Comfortable discussing with relatives	0.179	**0.760**	0.065
**Item 13** [10]—Feeling positive disclosing disability	0.076	**0.714**	0.081
**Item 21** [13]—Treat sibling naturally	0.071	**0.541**	**0.400**
**Item 14** [11]—Family not obliged to overextend	0.129	0.113	**0.790**
**Item 12** [9]—Parents invest more in siblings without disabilities	0.218	0.247	**0.757**
**Item 22** [14]—Less attention to a child with a disability is reasonable	0.256	0.036	**0.623**
**Explained variance**	**25.41%**	**16.29%**	**15.13%**

Factor loadings greater than 0.300 are in bold. Factor 3′s full label is Parental Attention to Typically-Developing Siblings' Needs.

The factor analysis revealed the existence of three factors. The first factor was labeled Burden and Limitation (items 1 [1], 2 [2], 3 [3], 7 [5], 9 [6], 10 [7], and 19 [12]) and showed a reliability of α = 0.85. The second factor was labeled Openness to Sharing (items 4 [4], 11 [8], 13 [10], and 21 [13]) and showed a reliability of α = 0.72. The third factor was labeled Parental Attention to TD Siblings' Needs (items 12 [9], 14 [11], and 22 [14]) and showed a reliability of α = 0.67. The internal consistency of the third factor (α = 0.67) was lower than the other two factors. Given that this subscale comprises only three items, its reliability should be interpreted cautiously, and observed associations involving this factor may be attenuated. Accordingly, results regarding this dimension are viewed as preliminary and in need of further refinement. Given that perceptions of parental attention and fairness are culturally and contextually embedded, future studies should replicate this factor's reliability and structure in diverse cultural and family contexts.

### 3.2 Confirmatory factor analysis

**Figure 1:**
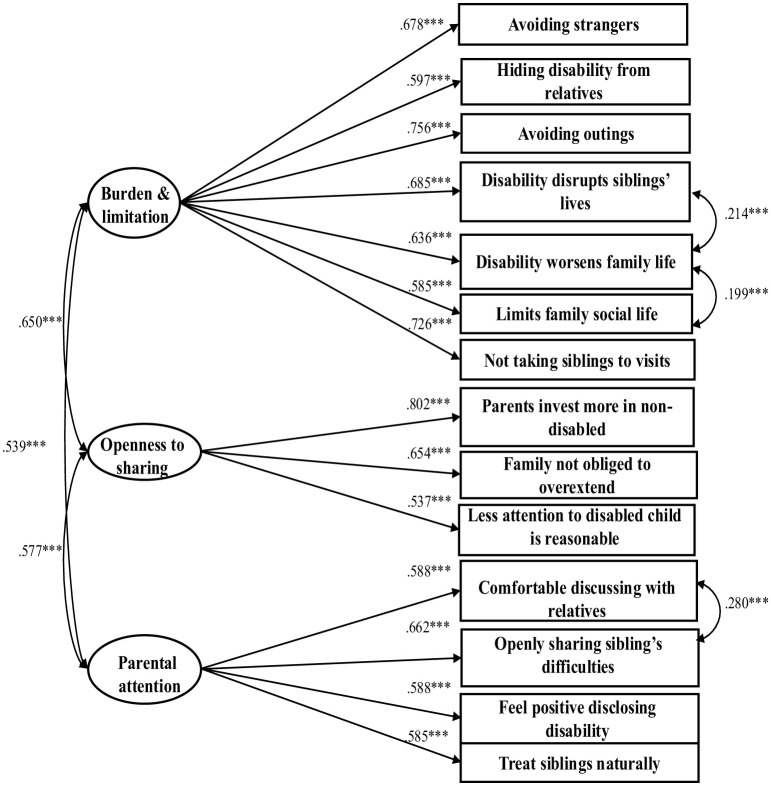
Confirmatory factor analysis (CFA) model of the Sibling Acceptance Questionnaire.

In order to further examine the questionnaire, we conducted a confirmatory factor analysis (CFA) on the factor structure that emerged from the EFA. The CFA was conducted using R software (Lavaan package). The model fit indices showed good fit to the data: χ^2^ (71) = 376.68, *p* < 0.001, χ^2^*/df* = 5.31, CFI = 0.923, TLI = 0.901, RMSEA [90% C.I.] = 0.071 [0.064, 0.078], SRMR = 0.047. The observed variables loaded significantly on the latent factors (see Table 3 and Figure 1).

**Table 3:** Standardized factor loadings for observed variables on latent constructs.

**Items**	**Latent factor**	**Loadings**
**Item 19** [12]—Avoiding strangers with sibling	Factor 1	0.676
**Item 1** [1]—Hiding disability from relatives		0.585
**Item 2** [2]—Avoiding outings with sibling		0.770
**Item 3** [3]—Disability disrupts siblings' lives		0.688
**Item 7** [5]—Disability worsens family life		0.644
**Item 9** [6]—Limits family social life		0.598
**Item 10** [7]—Not taking sibling to visits		0.709
**Item 12** [9]—Parents invest more in siblings without disabilities	Factor 2	0.788
**Item 14** [11]—Family not obliged to overextend		0.640
**Item 22** [14]—Less attention to a child with a disability is reasonable		0.520
**Item 4** [4]—Comfortable discussing with relatives	Factor 3	0.576
**Item 11** [8]—Openly sharing sibling's difficulties		0.663
**Item 13** [10]—Feeling positive disclosing disability		0.579
**Item 21** [13]—Treat sibling naturally		0.584

Table 4 presents descriptive statistics including means, standard deviations, and range (minimum and maximum values) for all questionnaire items. The results indicate that item means were generally above the scale midpoint on the 5-point scale (ranging from 1 to 5), while standard deviations demonstrated relatively consistent patterns across items.

**Table 4:** Theoretical statistics (means, SD, min/max) of the questionnaire items.

**Items**	***M***	***SD***	**Min**	**Max**
**Item 19**[12]—Avoiding strangers with sibling	3.58	1.30	1	5
**Item 1** [1]—Hiding disability from relatives	4.16	1.11	1	5
**Item 2** [2]—Avoiding outings with sibling	3.96	1.21	1	5
**Item 3** [3]—Disability disrupts siblings' lives	3.61	1.23	1	5
**Item 7** [5]—Disability worsens family life	3.84	1.18	1	5
**Item 9** [6]—Limits family social activities	3.39	1.28	1	5
**Item 10** [7]—Not taking sibling to visits	4.05	1.15	1	5
**Item 12** [9]—Parents invest more in siblings without disabilities	4.31	1.03	1	5
**Item 14** [11]—Family not obliged to overextend	4.18	1.11	1	5
**Item 22** [14]—Less attention to a child with a disability is reasonable	3.62	1.19	1	5
**Item 4** [4]—Comfortable discussing with relatives	3.89	1.27	1	5
**Item 11** [8]—Openly sharing sibling's difficulties	3.79	1.30	1	5
**Item 13** [10]—Feeling positive when disclosing sibling's disability	3.61	1.21	1	5
**Item 21** [13]—Treating sibling in a natural way	4.08	1.13	1	5

### 3.3 Differences in acceptance by functionality level

To examine whether there are differences by the sibling's functioning level (low/moderate/high) across the three acceptance measures (Burden and Limitation, Openness to Sharing, and Parental Attention to TD Siblings' Needs), three one-way ANOVAs were conducted. Examination of Table 5 reveals statistically significant differences by functioning level in Burden and Limitation and Openness to Sharing. Scheffe *post-hoc* analyses revealed that for both Burden and Limitation and Openness to Sharing, acceptance levels of siblings with high or moderate functioning were significantly higher than acceptance levels of siblings with low functioning, with no significant difference between acceptance levels of siblings with high or moderate functioning. In contrast, no statistically significant difference was found by functioning level in sibling acceptance according to Parental Attention to TD Siblings' Needs.

**Table 5:** Means and standard deviations of three acceptance factors by functionality levels.

**Functionality level**	**n**	***M***	***SD***	***F***
**Burden and limitation**				
High	311	3.91	0.83	
Moderate	462	3.80	0.88	
Low	79	3.33	0.94	*F* (2,849) = 13.82, *p* < 0.001, *η^2^* = 0.032
**Openness to sharing**				
High	311	4.10	0.82	
Moderate	462	4.06	0.85	
Low	79	3.61	0.99	*F* (2,849) = 11.12, *p* < 0.001, *η^2^* = 0.026
**Parental attention to TD siblings' needs**				
High	311	3.93	0.88	
Moderate	462	3.81	0.92	
Low	79	3.72	0.93	*F* (2,849) = 2.33, *p* = 0.098, *η^2^* = 0.005

TD, typically-developing.

### 3.4 Differences in the three acceptance factors according to disability

To examine whether there are differences in the three acceptance factors (Burden and Limitation, Openness to Sharing, and Parental Attention to TD Siblings' Needs) between siblings of individuals with ASD and Down syndrome, three independent sample t-tests were conducted. Examination of Table 6 reveals statistically significant differences by functioning level in all three acceptance measures. For both Burden and Limitation, Openness to Sharing, and Parental Attention to TD Siblings' Needs, acceptance levels of siblings of ASD were significantly higher than acceptance levels of siblings of Down Syndrome.

**Table 6:** Means and standard deviations of three acceptance factors by disability.

**Functionality level**	**n**	***M***	***SD***	***t***
**Burden and limitation**				
ASD	479	3.97	0.84	
Down syndrome	374	3.58	0.90	*t* (851) = 6.45, *p* < 0.001
**Openness to sharing**				
ASD	479	4.17	0.77	
Down syndrome	374	3.86	0.95	*t* (851) = 5.20, *p* < 0.001
**Parental attention to TD siblings' needs**				
ASD	479	3.97	0.83	
Down syndrome	374	3.68	0.97	*t* (851) = 4.51, *p* < 0.001

## 4 Discussion

The present study represents the first systematic validation of the Sibling Acceptance Questionnaire for use among typically-developing siblings of individuals with developmental disabilities. The instrument was originally developed in Hebrew by Brenner (1979) to measure parental acceptance of a child with a disability and has since been used with parents in several studies. A sibling version was later introduced by Koenig-Gorfinkel (2004), yet to date, no formal psychometric validation had been undertaken for this adaptation. By applying both exploratory and confirmatory factor analyses and by examining associations with the functional independence of the sibling with the disability, the current research provides important evidence for the construct validity, content validity, and reliability of the questionnaire.

### 4.1 Factorial structure of sibling acceptance

The analyses revealed a three-factor structure that reflects distinct aspects of sibling acceptance. The first dimension encompassed items relating to perceptions of burden and limitation in family and personal life. The second dimension captured openness to sharing, that is, the extent to which siblings feel comfortable discussing their brother's or sister's disability with others. The third dimension reflected perceptions of parental attention and the distribution of resources between the siblings with and without disabilities.

The first dimension that emerged from the questionnaire validation, perceptions of burden and limitations, refers to concrete challenges that siblings experience in their daily lives. This receives support from existing studies where siblings reported restrictions on shared activities such as going on vacations or eating out, along with financial pressures and parental stress, along with additional caregiving responsibilities and consequent earlier maturation (Linimayr et al., 2025). The challenges that they experience help shape some of the decisions that are made during emerging adulthood, regarding aspects of their lives such as career choices, places to live, and the like (e.g., Beffel et al., 2023). For example, more than half of participants in the study by Wright et al. (2024) reported that their sibling with autism influenced their career path, often leading them into health, education, and social service fields. Similarly, Lemoine and Schneider (2024) found that decisions about where to live were shaped by sibling relationships, particularly among women, who reported a stronger desire to remain close to their sibling with Down syndrome. Families in general were often described as “atypical” compared to their peers, a perception associated with feelings of difference and exclusion (Niedbalski, 2024). These findings suggest that perceptions of burden are reflected in clear limitations, responsibilities, and life trajectories, which aligns with the first dimension of the validated scale.

The second dimension validated in the questionnaire was openness to sharing, which highlights typically-developing siblings' willingness and ability to discuss their sibling's disability. Drawing from prior research, barriers to such openness include stigma, embarrassment, and cultural attitudes that foster feelings of shame or isolation (Jajodia and Roy, 2022). Stigma, shaped by societal perceptions of disability, disrupts siblings' sense of normality (Hwang and Charnley, 2010). Moreover, many siblings hesitate to disclose their own challenges to parents, aiming to avoid exacerbating parental stress (Múries-Cantán et al., 2023). Despite these obstacles, existing studies indicate that siblings still value and actively pursue close communication with their sibling with a disability. The present study demonstrates that this measure effectively captures siblings' perceptions of discussing their sibling's disability. For those who struggle with this aspect, support groups are now increasingly available for siblings of individuals with disabilities, which offer valuable resources. Sharing experiences in a “safe space” with peers facing similar challenges can provide meaningful support (e.g., Vatne and Zahl, 2017).

The third dimension, perceptions of parental attention and resource allocation, reflects siblings' awareness of how parents divide time and emotional resources between children. Many siblings have reported limited parental availability, as parents' energy was directed primarily toward the child with a disability (Levante et al., 2025). This imbalance often led to feelings of neglect and lack of support for the typically-developing child. Siblings also described parents exhibiting greater tolerance for the child with a disability and imposing more stringent expectations on those without disabilities. Furthermore, in many families, typically-developing siblings assumed additional responsibilities, reinforcing perceptions of injustice and frustration (Levante et al., 2025). Importantly, parents themselves often recognized this imbalance and expressed guilt over the unequal distribution of attention (Jajodia and Roy, 2022). These patterns were associated with negative emotional outcomes in the siblings, including anger, jealousy, and resentment, as well as externalizing behaviors such as defiance and risk-taking (Múries-Cantán et al., 2023). These findings suggest that perceptions of parental attention represent a central dimension of sibling acceptance, rooted in the broader family system. Yet, because norms regarding parental role expectations and the distribution of resources may vary across cultures and family structures, this subscale may be particularly sensitive to contextual differences, underscoring the need for cross-cultural validation. At the same time, this dimension showed somewhat lower internal consistency in the present study. This may reflect the brevity of the subscale and the complexity of capturing perceptions of parental resource allocation with a small number of items. Therefore, conclusions drawn from this factor should be treated with caution, and future studies should strengthen this subscale before using it for fine-grained comparisons or applied decision-making.

### 4.2 Functional independence of the sibling with disability

The findings of the current study indicate that sibling acceptance is closely linked to the functional independence of the brother or sister with a disability, that is, the ability of the sibling with the disability to independently manage day-to-day actions such as getting dressed, using transportation, and the like. When siblings described their brother or sister as having lower levels of independence, they also tended to report lower acceptance scores, particularly in the first two factors that captured the domains of burden and openness to sharing. These results echo earlier accounts that revealed that uncertainty, communication difficulties, and behavioral challenges intensify the stress experienced by siblings (Alon, 2025b; Trew, 2025). Similarly, research has demonstrated that limited verbal interaction, difficulties in emotional expression, and a tendency of the sibling with a disability to withdraw are associated both with siblings' heightened sense of burden and reduced willingness to share openly (Lee et al., 2019)—i.e., to both of the factors of the acceptance questionnaire. At the same time, siblings frequently have faced the social consequences of stigma and judgment from peers or the broader community, including embarrassment about their brother or sister and feelings of guilt or isolation that were reinforced by cultural attitudes (Múries-Cantán et al., 2023; Wright et al., 2024).

These dynamics can be further complicated when siblings live outside the family home. Students, for example, often reported that their contact with a brother or sister with ASD was mediated indirectly through other family members rather than established through direct communication, leaving them with a sense of distance or disconnection (Wright et al., 2024). This can be particularly relevant during emerging adulthood, when individuals are separating more from their family of origin.

Alongside these challenges, siblings expressed a strong need for opportunities to share experiences and to access reliable information. Many highlighted the importance of feeling understood by peers who also had siblings with intellectual or developmental disabilities, as these exchanges provided validation and reduced feelings of isolation (Múries-Cantán et al., 2023). Emotional support following diagnosis, particularly guidance on communication strategies and coping resources, was also identified as a key factor that could improve sibling relationships and enhance mutual understanding (Levante et al., 2025).

A significant finding was that the functional independence of the sibling with a disability was not related to the third dimension of the questionnaire, that of Parental Attention and Resource Allocation. This lack of association may be explained by the fact that perceptions of parental attention are shaped less by the functional abilities of the child with a disability and more by the broader dynamics of family life. Siblings' sense of fairness and support depends primarily on how parents divide their time, emotional resources, and expectations among their children, regardless of the specific level of independence of the child with a disability. In other words, even when a sibling demonstrates greater or lesser functional autonomy, what matters for the typically-developing sibling is their perception of whether their parents are sufficiently attentive and responsive. This finding highlights that the third dimension reflects systemic family processes rather than characteristics of the sibling with a disability, distinguishing it from the other two factors.

Taken together, these findings suggest that the level of functional independence of the sibling with a disability has a direct impact on how acceptance is experienced. Communication challenges, the weight of their responsibilities, and the influence of stigma, all contribute to shaping the everyday realities of sibling relationships. At the same time, the availability of supportive environments and access to relevant information can mitigate these difficulties and foster stronger, more resilient connections.

### 4.3 Limitations and future directions

Several limitations of the present study should be acknowledged. The exclusive reliance on self-report questionnaires introduces the possibility of bias due to socially desirable responding. To enhance interpretive depth, future studies should adopt multi-informant designs (e.g., parent reports and, when feasible, reports from the sibling with a disability, or other family members) to triangulate perceptions and reduce single-source bias. In addition, qualitative or mixed-method approaches (e.g., semi-structured interviews, focus groups, or reflective diaries) may enrich interpretation by contextualizing questionnaire scores and clarifying how acceptance is experienced and negotiated in everyday family life.

Next, an important methodological limitation of the present study concerns the use of the same sample for both exploratory (EFA) and confirmatory (CFA) factor analyses. Although this approach provided initial support for the factor structure of the sibling acceptance questionnaire, it is important to acknowledge that conducting CFA on the same data used for EFA can inflate model fit indices and increase the risk of overfitting. This occurs because the CFA essentially confirms patterns derived from the same data, rather than providing independent validation. Consequently, the confirmatory results should be interpreted with appropriate caution. Future research should replicate the proposed factor structure using an independent sample to provide more robust evidence of construct validity.

Regarding the factors themselves, although the internal consistency of the first two factors was high, indicating that the questionnaire reliably captures these constructs, the reliability of the third factor was somewhat lower. While still within an acceptable range for validation research, this result suggests that future refinement of the instrument could include the development of additional items to strengthen this dimension. Future refinement of this subscale could follow a multi-step process: (a) generate an expanded pool of items that captures different facets of perceived parental attention to typically-developing siblings (e.g., emotional availability, responsiveness to the TD sibling's concerns, perceived fairness, and recognition of the TD sibling's needs); (b) obtain qualitative feedback from typically-developing siblings (e.g., cognitive interviews, focus groups, or open-ended comments) to evaluate item clarity, sensitivity, and relevance; and (c) pilot-test the expanded item pool and conduct item analyses (e.g., item–total correlations and factor loadings) to retain the most informative items and re-evaluate reliability in an independent sample. Thus, although the current tool is sufficiently robust for research purposes, continued improvement would further enhance its precision and applicability. An additional limitation concerns construct coverage: acceptance is a complex construct that may also be expressed through related domains (e.g., emotional closeness, ambivalence, caregiving motivations, and resilience). Therefore, when a more comprehensive assessment is needed, we recommend using this instrument alongside complementary measures of sibling relationship quality and coping resources.

Other recommendations for future research include longitudinal designs, which can capture the variability and stability of sibling acceptance across key family transitions, such as parental aging or changes in caregiving responsibilities. Additionally, studies of predictive validity could examine whether acceptance scores are associated with meaningful outcomes, including psychological adjustment, relationship quality, or caregiving involvement. Expanding research to include more diverse populations and cultural contexts would also strengthen the generalizability of the findings and provide deeper insight into the dynamics of sibling acceptance. In particular, future studies should examine the *Parental Attention and Resource Allocation* subscale across different cultural groups and family constellations (e.g., single-parent and blended families) and test measurement invariance. Such work will clarify whether the factor functions similarly across contexts or whether culturally specific item refinements are needed. Future research also should conduct cross-cultural validation and test measurement invariance across cultural groups and family contexts.

### 4.4 Implications and conclusions

Despite these limitations, the study's findings lend themselves to a variety of clinical and practical applications. Clinically, the Sibling Acceptance Questionnaire can be incorporated into intake and ongoing assessment in family-centered services (e.g., developmental clinics, counseling centers, and community disability support programs) to identify typically-developing siblings who may be at risk for distress or relational strain. Patterns across the three subscales can inform case formulation and targeted support. For example, elevated Burden and Limitation scores may indicate a need for stress-management and coping-focused support and guidance around balancing caregiving responsibilities with emerging-adulthood developmental tasks. Low Openness to Sharing may signal heightened stigma concerns and avoidance, suggesting psychoeducation and stigma-sensitive interventions (e.g., communication skills, narrative approaches, or peer sibling support groups) to strengthen disclosure comfort and social support. Lower perceived Parental Attention to Typically-Developing Siblings' Needs may highlight a family-systems target, indicating the potential utility of parent guidance and family sessions aimed at improving the visibility of the typically-developing sibling's needs, strengthening parental responsiveness, and facilitating structured family communication.

From an intervention-development perspective, the questionnaire provides a practical tool for (a) screening and stratifying participants for tailored program components, and (b) evaluating intervention outcomes by using factor scores as pre–post indicators of change. For instance, sibling-group interventions could prioritize modules aligned with the dominant acceptance profile (e.g., burden-focused vs. openness-focused), while family-based interventions could monitor whether efforts to improve communication and perceived fairness translate into improved acceptance scores over time.

At the policy and service-planning level, routine assessment of sibling acceptance can support the inclusion of typically-developing siblings as recognized stakeholders in care planning and transition services (“life after the parents”), helping agencies allocate resources to sibling support, psychoeducation, and family counseling. Aggregated data may also inform needs assessments for community programs by identifying which acceptance domains are most challenged in specific subgroups (e.g., by disability type or functional independence level). In practice, the questionnaire can be paired with brief measures of sibling relationship quality (e.g., closeness/ambivalence) and resilience or caregiver motivation to triangulate needs and guide individualized intervention planning.

The current study offers several important strengths. It is based on a large and diverse sample, lending power and generalizability to the findings, and validates the instrument from multiple perspectives, including construct validity, content validity, and reliability. This contributes added weight to the multidimensional structure of acceptance and emphasizes that it is a measurable construct. By validating a measure that captures the various aspects of sibling acceptance, the present research can provide scholars and clinicians with a valuable tool for advancing both theory and practice.

## Data Availability

The original contributions presented in the study are included in the article/supplementary material, further inquiries can be directed to the corresponding author.

## Appendix A—Original 25-item Questionnaire (items included in the analysis appear with their numbers in square brackets)

[1] I think it is not advisable to tell relatives what isn't ok with my sibling.
[2] I think that one shouldn't go out with a sibling with disabilities to shop for groceries, go on trips, etc. because of the difficulties it causes.
[3] My sibling's presence at home bothers the other siblings.
[4] For me personally, I feel free to discuss my sibling's limitations with relatives.
[5] In my opinion, a sibling with a disability changes life at home for the worse.
[6] The sibling with the disability limits the family lives of all the family members.
[7] In my opinion, it's better not to take my sibling with the disability to visit relatives, friends, or neighbors because they're a burden.
[8] I can openly talk about the difficulties of my sibling with the disability.
[9] I think it's better that my parents will invest the most of their time in their non-disabled children as they will get better results.
[10] I feel better when I tell other people that my sibling has a disability.
[11] In my opinion, the family doesn't always have to do the maximum possible for a child with a disability.
[12] I avoid running into strangers when I am with my sibling with a disability.
[13] In my opinion, siblings should relate to their sibling with a disability in the more natural way.
[14] I think that in order to take good care of the other children, it makes sense for the mother to pay less attention to the child with the disability.
